# Laparoscopic detorsion of the ovary in ovarian hyperstimulation syndrome during the sixth week of gestation: A case report and review

**DOI:** 10.1016/j.ijscr.2019.04.051

**Published:** 2019-05-09

**Authors:** Seiji Kanayama, Hiroko Kaniwa, Masako Tomimoto, Bo Zhang, Kazuhiro Nishioka, Hidekazu Oi

**Affiliations:** Department of Obstetrics and Gynecology, Nara Hospital, Kinki University, Faculty of Medicine, 1248-1 Otuda-Chou, Ikoma, Nara, 630-0293, Japan

**Keywords:** OHSS, ovarian hyperstimulation syndrome, Ovarian hyperstimulation syndrome, Ovarian torsion, Laparoscopic detorsion

## Abstract

**Introduction:**

Ovarian torsion in ovarian hyperstimulation syndrome (OHSS) is a relatively rare but serious complication in pregnant women. A delay in treatment increases the risk for functional loss of the ovary and early termination of pregnancy. In this report, we present the case of a 40-year-old female with OHSS who experienced ovarian torsion that was successfully treated with laparoscopic detorsion.

**Presentation of case:**

A 40-year-old pregnant woman in the 6th week of gestation who had conceived following *in vitro* fertilization presented to us with severe and persistent lower abdominal pain. Ultrasound examination revealed a viable singleton intrauterine pregnancy and bilateral enlarged ovaries with scanty ascites. Approximately 14 h after symptom onset, exploratory laparoscopy was performed. The right ovary was found to be twisted once around over the pedicle, and laparoscopic detorsion was completed. Postoperative follow-up was uneventful, and she successfully delivered a healthy infant at 38 weeks of gestation.

**Discussion:**

Although the reports on successful laparoscopic surgery for pregnant women with ovarian torsion are becoming more frequent, there are few reports on laparoscopic surgery for ovarian torsion in OHSS during the early first trimester. Optimal management of ovarian torsion during pregnancy needs to be explored for these patients.

**Conclusion:**

Immediate explorative laparoscopic surgery is a potentially safe and useful strategy for treating ovarian torsion during the early first trimester of pregnancy.

## Highlights

• Delayed treatment & diagnosis of ovarian torsion may cause pregnancy termination.
• Optimal management for these patients remains unstandardized.
• No consensus exists regarding the appropriate surgical approach.
• A 40-year-old pregnant female presented to us with ovarian torsion and OHSS.
• She was successfully treated with laparoscopic detorsion.

## 1 Introduction

Ovarian torsion is a relatively common gynecological emergency and is the fifth most common gynecological surgical emergency [1,2]. While a diagnosis of ovarian torsion is likely to be missed due to its nonspecific symptoms, prompt surgical intervention is needed to preserve ovarian function and fertility. A delay in the diagnosis and treatment of ovarian torsion in pregnant women can lead to early termination of pregnancy. However, optimal management for these patients remains unstandardized, and no consensus exists regarding the appropriate surgical approach. Recently, several institutions have reported the usefulness and safety of explorative laparoscopic surgery for the management of ovarian torsion during pregnancy [[3], [4], [5], [6], [7]], but most cases described in the above reports occurred around 10 weeks or later, and there are few reports on laparoscopic surgery for ovarian torsion in OHSS during the early first trimester. Optimal management of ovarian torsion during pregnancy continues to be explored. Here we describe the case of 40-year-old female who had conceived following *in vitro* fertilization and presented to us with ovarian torsion and OHSS during the 6th week of gestation. She was successfully treated with laparoscopic detorsion. This work has been reported according to the SCARE criteria [8].

## 2 Presentation of case

A 40-year-old female who had conceived following *in vitro* fertilization presented to an antenatal clinic in the 6th week of gestation complaining of acute lower abdominal pain.

At the same clinic, she had received *in vitro* fertilization treatment with a long gonadotropin-releasing hormone agonist protocol to induce ovarian stimulation for unexplained secondary infertility. She was referred to our hospital due to severe and persistent lower abdominal pain. Her medical history was unremarkable. Her physical examination revealed right lower abdominal tenderness without muscle guarding. Transvaginal ultrasound examination revealed a viable singleton intrauterine pregnancy.

**Fig. 1:**
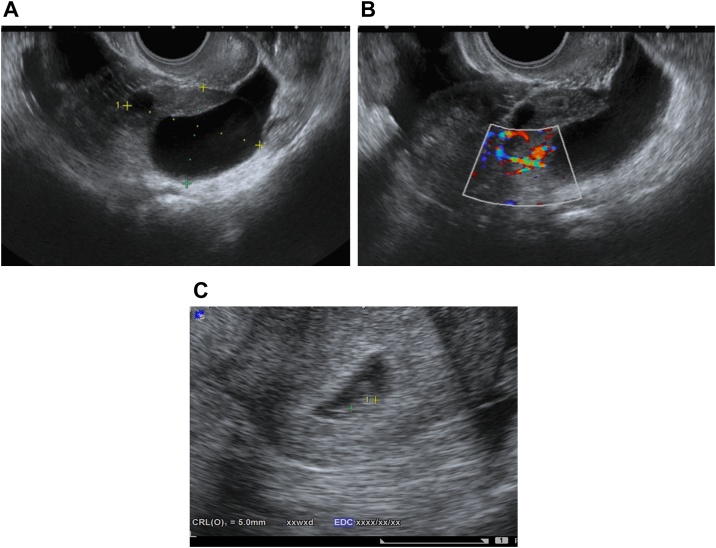
(A–C) Transvaginal ultrasound examination in the 6th week of gestation revealed a viable, singleton intrauterine pregnancy. The crown-rump length was 5.0 mm, and the patient had bilateral enlarged ovaries (right: 6.9 cm × 4.5 cm, left: 4.5 cm × 3.2 cm) with scanty ascites. Doppler ultrasonography revealed normal ovarian blood flow.

**Fig. 2:**
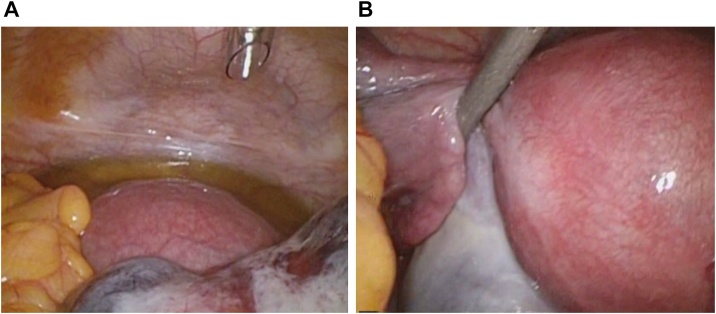
(A) A small amount of ascites was present, and the uterus appeared about normal large and shape. (B) The left ovary was cystic and slightly enlarged (4 cm × 3 cm) but appeared intact, with no evidence of torsion.

**Fig. 3:**
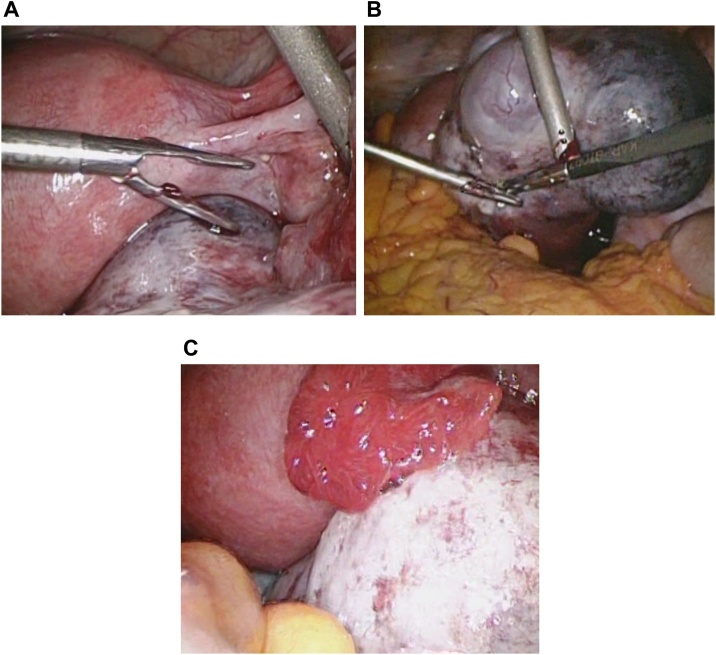
(A–D) The right ovary was twisted once around over the pedicle and was 6.4 cm × 4.0 cm in diameter. The right ovarian wall was twisted and showed hemorrhagic and congestive changes but no necrosis. Laparoscopic detorsion was performed carefully, and the twisted right ovary was released with relative ease. The ovary’s ischemic bluish color improved quickly.

The crown-rump length was 5.0 mm, and the patient had bilateral enlarged ovaries (right: 6.9 cm × 4.5 cm, left: 4.5 cm × 3.2 cm) with scanty ascites. Doppler ultrasonography revealed normal ovarian blood flow (Fig. 1A–C). She was diagnosed with mild OHSS, and torsion of the enlarged right ovary was suspected. Approximately 14 h after the onset of her acute abdominal symptoms, explorative laparoscopy was performed. The uterus appeared normal in size and shape (Fig. 2A). The left ovary was cystic and slightly enlarged with no evidence of torsion (Fig. 2B). The right ovary was twisted once around over the pedicle and was 6.4 cm × 4 cm in diameter; it showed hemorrhagic and congestive changes, but no necrosis. Laparoscopic detorsion was completed, and the twisted right ovary was released with relative ease. The ischemic bluish color of the ovary improved quickly (Fig. 3A–D). Because the congestive ovarian tissue was fragile and easy to tear, we did not perform oophoropexy in this case. The patient’s intra-abdominal pressure was 10 mmHg. The operative blood loss was small, and the operative time was 46 min. There were no intraoperative complications, and postoperative follow-up was uneventful. She successfully delivered a healthy infant at 38 weeks’ gestation. Follow-up ultrasound showed a normally functioning ovary. The patient’s consent was obtained to report this case, and the report was exempted from ethical approval by our institution.

## 3 Discussion

Ovarian torsion is an acute condition, and a delay in treatment increases the risk of functional loss of the ovary and early termination of the pregnancy; therefore, prompt diagnosis and surgical treatment are essential. Classical risk factors for ovarian torsion such as a large tumor, pregnancy, and ovarian hyperstimulation for the treatment of infertility are well known [2]. The symptoms of ovarian torsion are usually nonspecific; however, acute abdominal pain occurs in many cases, often starting suddenly and persisting for more than 24 h. Ultrasonography is the most safe and useful imaging modality for a prompt and accurate diagnosis in pregnant females with acute abdominal pain due to its wide availability and absence of radiation. Ultrasonographic findings in patients with ovarian torsion include a cystic mass, with or without pelvic fluid, intra-cystic hemorrhage; and thickening of the cystic wall due to edema or vascular and lymph engorgement [9].

However, these findings are not specific to ovarian torsion, and the condition can be difficult to distinguish from diseases such as tubo-ovarian abscess and hemorrhagic ovarian cysts. Several studies have demonstrated the diagnostic utility of color Doppler sonography [10,11]. In our case, Doppler ultrasonography revealed normal ovarian blood flow, and the twisted and swollen ovary was not macroscopically necrotic during laparoscopic surgery. Therefore, attention must be paid to blood flow parameters within the twisted vascular pedicle, as these measures are helpful for predicting viable ovarian function; however, as demonstrated by the present case, the diagnosis of torsion should not be excluded if blood flow is present.

As a classical standard surgical procedure, adnexectomy has been conventionally performed to avoid the risk of thromboembolism after detorsion. However, several studies have reported found that compared with adnexectomy of the twisted adnexa, detorsion does not increase the risk for pulmonary embolism. At present, detorsion is regarded as a relatively safe treatment for reproductive-aged females or children, even those with twisted ischemic adnexa [12,13]. Recently, there have been increasing reports of successful laparoscopic surgery for ovarian torsion, even in pregnant women [[4], [5], [6], [7],^14^,^15^]. However, these data are limited to case reports or small-sized retrospective studies; thus, additional case reports and long-term follow-up reports are needed to establish the optimal management for these patients.

Conservative management through detorsion carries a risk for ovarian torsion recurrence. Therefore, long-term follow-up is required to confirm treatment outcomes. Rackow et al. [16] have reported a case of unilateral torsion and subsequent adnexal torsion in a pregnant female with OHSS. They performed laparoscopic detorsion for right adnexal torsion at 7 weeks’ gestation; however, subsequently, contralateral adnexal torsion occurred at 19 weeks, which was managed with emergency laparotomy with additional salpingo-oophorectomy.

Although preventing recurrent adnexal torsion is a clinically important management strategy, indications and approaches remain non-standardized due to the rarity of this condition. There have been several reports on the utility of preventive surgical techniques for recurrent torsions; oophoropexy is one of these techniques, and it involves fixation of the ovary to the pelvic sidewall, to the lateral round ligaments, or to the uterosacral ligament [[17], [18], [19], [20]]. Djavadian et al. noted that detorsion should be performed in the first step, but in case of recurrence, they recommend an oophoropexy to avoid reappearance of the torsion [18].Weitzman VN reported shortening of the utero-ovarian ligament as an alternative to oophoropexy to prevent recurrence [19].

## 4 Conclusions

Immediate explorative laparoscopic surgery might offer a potentially safe and useful strategy for treating ovarian torsion in OHSS during early pregnancy. However, to date, few case reports and small-sized retrospective studies have been reported on this topic. Thus, accumulation of additional case reports is needed to establish the prognostic significance and optimal management for such patients.

## Conflicts of interest

All authors declare that there are no conflicts of interest regarding the publication of this paper.

## Sources of funding

This research did not receive any specific grant from funding agencies in the public, commercial, or not-for-profit sectors.

## Ethical approval

The report was exempted from ethical approval by our institution.

## Consent

The patient’s consent was obtained to report this case, and the report was exempted from ethical approval by our institution.

## Author’s contribution

Study conception and design: Seiji Kanayama.

Acquisition of data: Hiroko Kaniwa and Masako Tomimoto.

Analysis and interpretation of data: Bo Zhang and Kazuhiro Nishioka.

Drafting of manuscript: Seiji Kanayama.

Critical revision: Hidekazu Oi.

## Registration of research studies

NA.

## Guarantor

Seiji Kanayama.

## Provenance and peer review

Not commissioned, externally peer-reviewed.
